# Impact of the introduction of drug eluting stents on clinical outcomes in patients undergoing percutaneous and surgical coronary artery revascularisation procedures in Western Australia

**DOI:** 10.1186/1471-2261-13-47

**Published:** 2013-07-05

**Authors:** Frank M Sanfilippo, Jamie M Rankin, Michael ST Hobbs, Michael Nguyen, Matthew W Knuiman, Patricia Berg, Eric G Whitford, Randall Hendriks, Bernard E Hockings, Michael Muhlmann, Mark Newman, Robert Larbalestier, Ian Gilfillan, Thomas G Briffa

**Affiliations:** 1 School of Population Health, The University of Western Australia, Crawley, Western Australia; 2 Department of Cardiology, Royal Perth Hospital, Perth, Western Australia; 3 Department of Cardiology, Fremantle Hospital, Fremantle, Western Australia; 4 Cardiology Department, Sir Charles Gairdner Hospital, Nedlands, Western Australia; 5 Coastal Cardiology, Murdoch, Western Australia; 6 Heart Care Western Australia, Perth, Western Australia; 7 School of Medicine and Pharmacology, University of Western Australia, Nedlands, Western Australia; 8 Department of Cardiothoracic Surgery, Sir Charles Gairdner Hospital, Nedlands, Western Australia; 9 Department of Cardiothoracic Surgery, Royal Perth Hospital, Perth, Western Australia; 10 Department of Cardiothoracic Surgery, Fremantle Hospital, Fremantle, Western Australia

**Keywords:** Coronary artery disease, Coronary revascularisation, Clinical outcome, Population study, Drug eluting stents, Percutaneous coronary intervention

## Abstract

**Background:**

Increasing rates of percutaneous coronary intervention (PCI) and decreasing rates of coronary artery bypass graft (CABG) surgery followed the introduction of drug eluting stents in Western Australia in 2002. We assessed the impact of these changes on one-year outcomes for the total population of patients undergoing coronary artery revascularisation procedures (CARP) in Western Australia between 2000-2004.

**Methods:**

Clinical and linked administrative data (inpatient admissions and death) were merged for all patients who had their first CARP with stent or CABG in Western Australia between 2000-2004. The clinical data were collected from all hospitals in Western Australia where CARP procedures are performed. We calculated the unadjusted (Kaplan-Meier) and adjusted (Cox) risks for one-year death (all-cause), death (all-cause) or admission for myocardial infarction (MI), target vessel revascularisation (TVR) and the composite outcome of death/MI/TVR (major adverse cardiac events, MACE).

**Results:**

Over the study period, there were 14,118 index CARPs. The use of drug eluting stents increased from 0% to 95.8% of PCI procedures, and PCI procedures increased from 61.1% to 74.4% of all CARPS. There were no temporal changes in adjusted one-year mortality or death/MI. Overall, adjusted one-year MACE fell from 11.3% in 2000 to 8.5% in 2004 (p<0.0001) due to a significant reduction in TVR in the PCI group.

**Conclusion:**

The introduction of drug eluting stents and resulting changes in coronary revascularisation strategies were not associated with changes in the one-year risk of major clinical endpoints (death or death/MI), but were associated with a significant reduction in the risk of MACE, driven entirely by a reduction in TVR after PCI. This real world study supports the effectiveness of drug eluting stents in reducing repeat procedures in the total CARP population without increasing the risk of death or MI.

## Background

Coronary artery revascularisation procedures (CARP) using percutaneous coronary intervention (PCI) or surgical (coronary artery bypass graft, CABG) techniques are commonly performed for patients with significant obstructive coronary artery disease to relieve angina, improve survival or both. Over the last 30 years, there has been stepwise improvement in revascularisation techniques, with a significant increase in the proportion of patients treated percutaneously [1]. Medical therapies have also improved significantly over this period. It is often difficult to assess the relative effectiveness and real-world safety of new techniques, with guidance usually coming from case series, registries and randomised controlled trials. Most of these studies examine either PCI or CABG or, in some cases, direct comparisons between different revascularisation strategies in selected populations [2-4]. Another method for assessing the impact of a new therapy is to examine population-based outcomes as the new therapy is introduced to an otherwise stable population. Such a study demonstrated improved outcomes following PCI as coronary stents were introduced into British Columbia in the mid-1990s [5].

As a new PCI technology, drug eluting stents (DES) became widely used in Western Australia from mid-2002 following clinical trials demonstrating the efficacy of DES in reducing clinical events [6,7]. This provided us with a unique opportunity to examine the real-world impact of DES by linking routinely collected State-wide health administrative data from the Western Australian Data Linkage System [8] with clinical registries covering all coronary revascularisation procedures. Our intention was to assess the impact of changing revascularisation strategies on outcomes following the release of DES in the total population of patients undergoing coronary artery revascularisation procedures, rather than to directly compare DES or PCI with CABG.

## Methods

### Participating hospitals and study population

In Western Australia (population 1.95 million in 2003), CARPs are carried out exclusively in the three adult teaching hospitals and in four private hospitals, all located in the Perth metropolitan area. Loss of cases to Centres in other States (located at least 2000 km distant) is considered to be very rare. The study population consisted of all patients who had a PCI or CABG procedure at any of these Perth hospitals during 2000 to 2005. Patients with out-of-State postcodes were excluded. There was no restriction on age. The PCI cases were limited to those who had stents, divided into procedures for bare metal stents (BMS) only or DES (with or without BMS). The cohorts included patients with left main disease as well as multi-vessel disease.

### Data sources and variables

Clinical data for PCI procedures were obtained from the seven hospitals and linked to records of hospital admissions (Hospital Morbidity Data Collection, HMDC) and death, which are two of the core datasets of the Western Australian Data Linkage System [8]. Records were matched using the patient’s unique medical record number, procedure date and hospital identifier code. CABG procedures in 2000-2005 were identified from the HMDC using codes from blocks 672-679 in the International Classification of Diseases (ICD) 10^th^ revision Australian Modification. The data included the number of vessels grafted and whether venous or arterial grafts were used. The CABG file was combined with the PCI file to provide a dataset of total CARPs for the 6-year period 2000-2005.

Details of PCI procedures from private hospitals were collected from billing sheets, computer databases (if available), medical notes (cardiologist or hospital notes), radiographer’s journal, nursing intervention records, as well as the cardiologist’s operation notes. Data from teaching hospitals were obtained from clinical databases maintained by cardiology departments with their own internal data checking procedures. Missing or inconsistent data were queried with cardiac catheter laboratory personnel of the corresponding hospital, and amended where possible.

The aggregated minimum dataset of PCI and CABG cases included: residential postcode, age, gender, admission and discharge dates, type of hospital, type of admission, discharge diagnosis and procedure codes (for current diagnosis and medical history), procedure date and time, vessels treated, number of grafts, type of grafts used, number and details of stents inserted (name, diameter, length), and date and cause of death.

### Treatment groups

Patients were classified as either index PCI or index CABG in 2000-2004 by applying a fixed look-back period of 5 years to the linked HMDC records to identify the first admission for PCI or CABG in 2000-2004. We limited the index cohort to 2000-2004 so that each person would have at least 12 months of follow-up. If a patient had both a PCI and CABG in the same index admission (n=52), we assigned them according to which procedure came first, and excluded 22 cases who had balloon angioplasty first or were missing data for the PCI type, leaving 30 cases with PCI (stents) plus CABG. The cohort is part of a current long-term follow-up, and here we report results of outcomes/endpoints in the first year of follow-up. Rather than comparing PCI and CABG, our study has investigated the total revascularisation cohort to determine whether the introduction of a new revascularisation technology has changed outcomes in the whole population of patients who had these procedures.

### Follow-up and outcomes

Outcomes and endpoints were assessed over one year for index CARP cases and included: (i) all-cause death; (ii) all-cause death or admission for MI (whichever was first); (iii) target vessel revascularization (TVR); and (iv) major adverse cardiac events (MACE) defined as all-cause death or MI admission or TVR, whichever was first. For index PCI cases, a TVR endpoint was defined as a subsequent PCI procedure on the same vessel as in the index PCI (as identified from clinical data), or as any subsequent CABG procedure, whichever was first. For index CABG cases, a TVR endpoint was any subsequent PCI or CABG procedure. Follow-up ceased on the date of an outcome/endpoint within 12 months of the index date or at the end of the 12-month period. Patients were censored if the outcome/endpoint did not occur during follow-up. There were too few one-year stroke events (91 in total) for a separate analysis of this outcome over time in any of the revascularisation cohorts. We have also reported 28-day mortality as part of the characteristics of the index admissions in the total revascularisation cohort.

### Comorbidities

The HMDC was used to identify previous admissions within 5 years of the index CARP where the discharge diagnosis was recorded as MI, stroke, diabetes, renal failure, congestive heart failure, peripheral vascular disease or chronic obstructive pulmonary disease in any diagnosis field. Similarly, a Charlson comorbidity score [9] was calculated for each index case by applying a fixed 5-year look-back period using the HMDC, excluding MI in the calculation of the score if it occurred in the index CARP admission. We used the Dartmouth-Manitoba ICD code assignments [10] in calculating the Charlson score based on the original 17 Charlson comorbidities.

### Statistical methods

To show the changing trends and uptake of all CARPs in Western Australia during 1980-2005, we plotted the age-standardised rates of admission for CARPs in males aged 35 years or older (males and females had similar trends). Rates were age-standardised by the direct method using the age distribution of the Western Australian resident population at 30 June 2001 (a census year) as the standard [11].

The Charlson comorbidity score was compared between index PCI and CABG using the Wilcoxon two-sample test with Normal approximation and continuity correction. Counts of one-year outcomes/endpoints are presented for each treatment group by year of index admission, and one-year unadjusted risks were estimated from corresponding Kaplan-Meier survival probabilities at 12 months. Temporal changes in unadjusted risks were assessed using the two-sided log-rank test across years.

Cox proportional hazards regression was used to estimate the adjusted risk for the outcomes/endpoints over the first 12 months by index year of admission separately for CABG, PCI and total CARPs. The models were adjusted for age, gender, Charlson comorbidity score, principal diagnosis at index admission, type of index admission (emergency, booked), type of hospital at index admission, number of vessels treated (single, multiple), and type of CARP (for the All CARPs models only). The year of admission variable in all Cox models was tested for and passed the proportional hazards assumption indicating that the relative risk of the outcome/endpoint did not change over the 12 month follow-up period for the index years. The same models were used to estimate the adjusted hazard ratio (HR) and 95% confidence interval for year of index admission and each adjustment variable in the total CARP cohort, and to plot adjusted survival curves for the outcome of MACE.

### Ethics approval

The study complies with the Declaration of Helsinki, and was approved by human research ethics committees of participating hospitals, the University of Western Australia and the Confidentiality of Health Information Committee of the Western Australian Department of Health. Being a large population-based epidemiological study, the approval included a waiver of informed consent.

## Results

### Trends in revascularisation procedures

**Figure 1:**
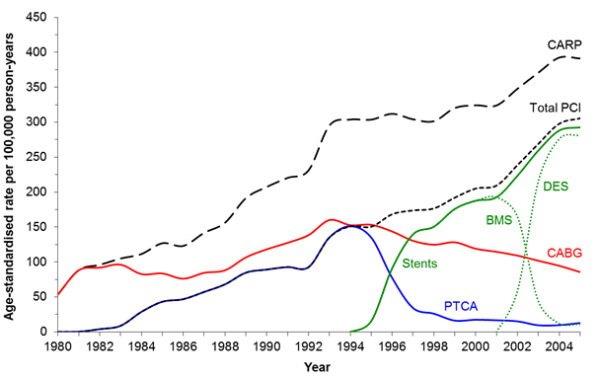
**Age-standardised rates of admission for coronary artery revascularisation procedures in males aged 35 years or older in Western Australia during 1980-2005.** CARP = total PCI + CABG; Total PCI = Stents + PTCA; PTCA, percutaneous transluminal coronary (balloon) angioplasty. Curves for females show a similar pattern. Although stents were available in Western Australia in 1988, there was little uptake until 1994, and there was no ICD-9-CM code until July 1995.

Figure 1 provides context to the long-term trends in coronary revascularisation in Western Australia since the introduction of CABG in the late 1970s and PCI in 1983. CARP rates increased throughout, with rates slowing from 1993, but increasing again from 1998. Rates of PCI surpassed those for CABG in 1994 after which the latter steadily declined. BMS were available from 1992 in Western Australia and rapidly replaced percutaneous transluminal coronary (balloon) angioplasty (PTCA). DES were introduced in mid-2002 (25% of PCI procedures) and largely replaced BMS by 2004 (95% of PCI procedures). This was accompanied by a further noticeable increase in the rate of PCI.

### Demographic and clinical characteristics

Table 1 shows demographic and clinical information for the 14,118 patients with index CARPs, consisting of 5174 patients with BMS, 4419 with DES and 4525 with CABG. The total count of procedures increased from 2669 in 2000 to 3088 in 2004, with much of the increase attributable to an increase in emergency admissions or in cases with a principal diagnosis of MI. However, there was a decline in the proportion of cases with a 5-year history of admission for MI. The total cohort had a mean age of 64 years, 76% were male, and persons undergoing CABG were significantly older and more likely to be male than those receiving PCI. Comorbidity, as measured by the Charlson score, did not change much over time, but CABG cases had a higher mean Charlson score than PCI cases. The majority of cases overall (67%) were single vessel disease, but only 25% of the CABG cohort had single-vessel disease compared with 86% in the PCI cohort. Arterial grafts were used slightly more than venous grafts in all years from 2000-2004, and the majority of CABG patients had both venous and arterial grafts used in the procedure. Deaths within 28 days of index admission occurred in 2% of the CARP cohort, with the proportion higher for CABG procedures than PCI (3.2% versus 1.4%) and lowest for DES procedures (1.1%). The majority of procedures were in public hospitals and consisted mainly of booked admissions rather than emergency.

**Table 1:** Patient characteristics at index admission for PCI (excluding angioplasty only) and CABG during 2000-2004

	**2000**	**2001**	**2002**	**2003**	**2004**	**Total**
Number of patients (col%)	2669	2652	2765	2944	3088	14118
BMS	1630 (61.1)	1725 (65.0)	1370 (49.6)	353 (12.0)	96 (3.1)	5174
DES ^¶^	0	0	486 (17.6)	1733 (58.9)	2200 (71.2)	4419
CABG	1039 (38.9)	927 (35.0)	909 (32.9)	858 (29.1)	792 (25.6)	4525
Age						
mean (SD)	63.7 (11.1)	63.8 (11.1)	64.2 (11.7)	64.0 (11.8)	64.3 (11.6)	64.0 (11.5)
range	27-93	30-92	27-94	26-93	30-93	26-94
Males (%)	76.0	77.0	76.4	76.6	75.6	76.3
Main diagnosis, MI (%) ^†^	550 (20.6)	588 (22.2)	798 (28.9)	901 (30.6)	998 (32.3)	3835 (27.2)
Prior disease, no. (%) ^‡^						
MI	572 (21.4)	511 (19.3)	532 (19.2)	583 (19.8)	509 (16.5)	2707 (19.2)
Stroke	51 (1.9)	41 (1.6)	37 (1.3)	30 (1.0)	44 (1.4)	203 (1.4)
Diabetes	434 (16.3)	469 (17.7)	493 (17.8)	572 (19.4)	558 (18.1)	2526 (17.9)
Renal failure	72 (2.7)	71 (2.7)	84 (3.0)	124 (4.2)	114 (3.7)	465 (3.3)
Congestive heart failure	222 (8.3)	182 (6.9)	192 (6.9)	225 (7.6)	191 (6.2)	1012 (7.2)
COPD	223 (8.4)	204 (7.7)	163 (5.9)	149 (5.1)	132 (4.3)	871 (6.2)
PVD	156 (5.8)	156 (5.9)	126 (4.6)	174 (5.9)	144 (4.7)	756 (5.4)
Charlson score (mean) ^§^	1.38	1.36	1.31	1.39	1.31	1.35
Number of vessels treated, no. (%) ^††^						
single	1688 (65.4)	1704 (66.3)	1829 (67.6)	1954 (67.9)	2047 (68.2)	9222 (67.1)
multi-vessel	891 (34.6)	867 (33.7)	876 (32.4)	925 (32.1)	956 (31.8)	4515 (32.9)
Patients with venous grafts in CABG cohort, n (%) ^#^	853 (82.1)	755 (81.5)	746 (82.1)	722 (84.2)	675 (85.2)	3751 (82.9)
Patients with arterial grafts in CABG cohort, n (%) ^#^	924 (88.9)	816 (88.0)	795 (87.5)	768 (89.5)	712 (89.9)	4015 (88.7)
Deaths within 28 days, no. (%)	53 (2.0)	45 (1.7)	58 (2.1)	41 (1.4)	80 (2.6)	277 (2.0)
Hospital type: public, no. (%)	1728 (64.7)	1668 (62.9)	1674 (60.5)	1791 (60.8)	1828 (59.2)	8689 (61.6)
Admission type: emergency, no. (%)	1008 (37.8)	1013 (38.2)	1100 (39.8)	1223 (41.5)	1366 (44.2)	5710 (40.4)

*CABG* coronary artery bypass graft surgery, *PCI* percutaneous coronary intervention, *BMS* bare metal stent, *DES* drug eluting stent; col%, column percent, *SD* standard deviation, *MI* myocardial infarction.

¶ DES are with or without BMS, and were first used in mid-2002 in Western Australia.

† Significant increasing trend during 2000-2004 in the proportion of MI as main discharge diagnosis (p<0.0001; Cochran-Armitage trend test).

‡ History of admissions for disease within 5 years of index admission for CABG or PCI. COPD, chronic obstructive pulmonary disease; PVD, peripheral vascular disease.

§ Charlson comorbidity score based on comorbidities within previous 5 years up to and including index admission, but excluding MI in the index admission. Mean Charlson score significantly higher in index CABG patients (p<0.0001 for each year; Wilcoxon two-sample test using Normal approximation with continuity correction).

†† For CABG these were identified from ICD codes for procedures recorded in the Hospital Morbidity Data Collection. Some data were missing for both PCI and CABG.

^#^ Venous grafts are saphenous or other vein grafts; arterial grafts are internal mammary artery (left or right), radial artery or epigastric artery grafts.

### Outcomes

**Figure 2:**
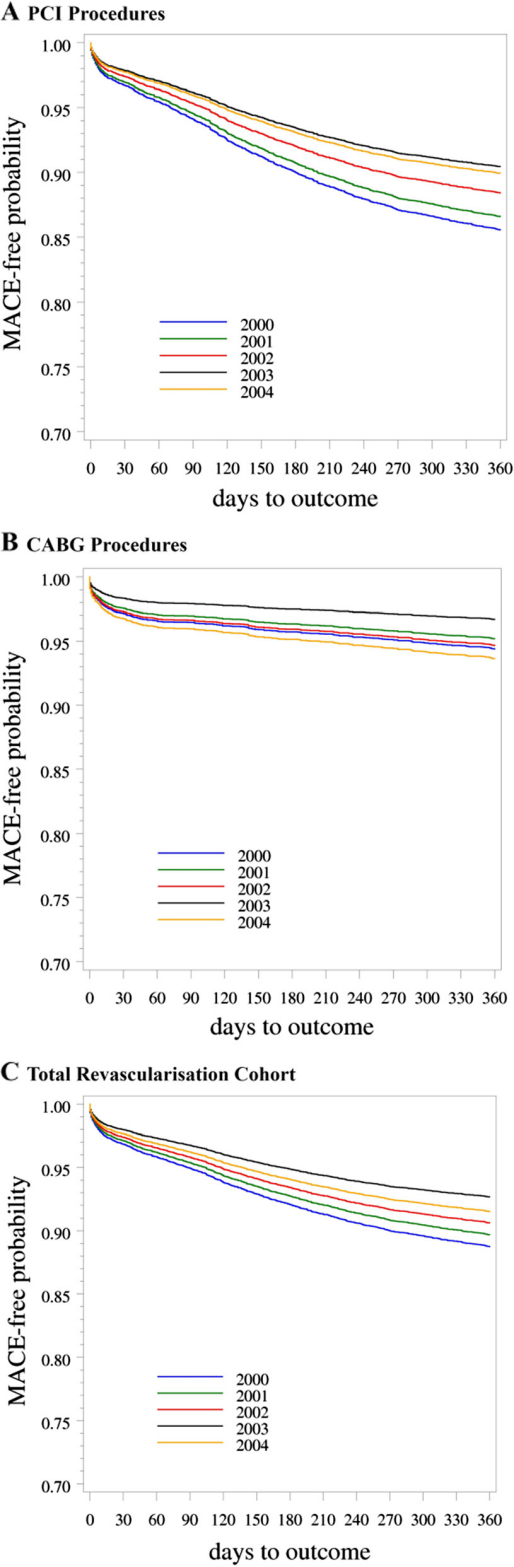
**Cox adjusted survival curves by year 2000-2004 showing the one-year outcome of MACE (major adverse cardiac events) in the revascularisation cohort (A-C).** The adjusting variables were age, gender, Charlson comorbidity score, principal diagnosis at index admission, type of index admission (emergency, booked), type of hospital at index admission, number of vessels treated (single, multiple), and type of CARP (for the All CARP models only).

Trends in adverse outcomes/endpoints (death, MI, TVR) within 12 months of index CARP are shown in Table 2 for each type of procedure, including estimates of one-year risk from Kaplan-Meier curves (unadjusted) and Cox regression models (adjusted), with corresponding Cox adjusted survival curves shown in Figure 2. For all CARP, there were no changes over time in the risk of major clinical endpoints (death or death/MI), but there was a marked and significant decline in TVR reflected also in the composite endpoint (MACE). The risk of death was higher in the CABG cohort than PCI, but risks were similar for death/MI, with no consistent trends over time. The PCI cohort had much higher unadjusted and adjusted risks for the technical endpoint (TVR) compared to the CABG cohort. However, the risk of TVR following PCI halved from 2000 to 2004 (p<0.0001) during the transition from BMS to DES. The risk of TVR following CABG was low and did not change over time. The unadjusted and adjusted risks of MACE declined significantly for the PCI group, but there was no clear trend in the CABG group. Overall for CARPS, there was a significant decline in unadjusted and adjusted risk of MACE over time which was driven by the decline in the PCI group. These results did not change after we excluded the 30 cases who had PCI (stents) plus CABG in their index admission. Whilst the survival curves (Figure 2) showed a gradual decline in the PCI group throughout the one-year follow-up, events were more likely to occur in the first 30 days in the CABG group. These analyses were repeated in the sub-cohorts of patients with multi-vessel disease. Trends in 12-month outcomes were similar to those of the total PCI and CABG cohorts reported in Table 2, with higher unadjusted and adjusted risks seen in the PCI subcohort with multi-vessel disease, and slightly higher risks in the CABG subcohort with multi-vessel disease (see Additional file 1: Table S2).

**Table 2:** One-year outcomes/endpoints by calendar year for 2000-2004 by type of revascularisation

	**2000**	**2001**	**2002**	**2003**	**2004**	**p-value**
**ALL CARPS (PCI, CABG)**						
Number of events (unadjusted one-year risk in %)*						
Death	104 (3.9)	101 (3.8)	111 (4.0)	98 (3.3)	139 (4.5)	0.22
Death/MI	164 (6.1)	152 (5.7)	166 (6.0)	151 (5.1)	205 (6.6)	0.15
TVR	193 (7.4)	190 (7.3)	154 (5.7)	136 (4.7)	145 (4.8)	<0.0001
MACE	331 (12.4)	312 (11.8)	294 (10.6)	260 (8.8)	320 (10.4)	0.0002
Adjusted one year risk in %**						
Death	2.3	2.2	2.4	2.0	2.7	0.18
Death/MI	4.6	4.1	4.4	3.7	4.8	0.12
TVR	6.1	5.8	4.4	3.4	3.3	<0.0001
MACE	11.3	10.4	9.4	7.4	8.5	<0.0001
**PCI**						
Number of events (unadjusted one-year risk in %)*						
Death	46 (2.8)	52 (3.0)	63 (3.4)	74 (3.5)	95 (4.1)	0.18
Death/MI	95 (5.8)	99 (5.7)	107 (5.8)	123 (5.9)	157 (6.8)	0.50
TVR	180 (11.2)	176 (10.4)	145 (8.0)	127 (6.2)	129 (5.8)	<0.0001
MACE	251 (15.4)	248 (14.4)	228 (12.3)	224 (10.7)	257 (11.2)	<0.0001
Adjusted one year risk in %**						
Death	1.8	1.9	2.0	1.9	2.3	0.67
Death/MI	4.5	4.4	4.3	4.1	4.9	0.69
TVR	11.0	10.3	7.9	6.0	5.6	<0.0001
MACE	14.5	13.5	11.6	9.6	10.1	<0.0001
**CABG**						
Number of events (unadjusted one-year risk in %)*						
Death	58 (5.6)	49 (5.3)	48 (5.3)	24 (2.8)	44 (5.6)	0.04
Death/MI	69 (6.6)	53 (5.7)	59 (6.5)	28 (3.3)	48 (6.1)	0.02
TVR	13 (1.3)	14 (1.6)	9 (1.0)	9 (1.1)	16 (2.1)	0.34
MACE	80 (7.7)	64 (6.9)	66 (7.3)	36 (4.2)	63 (8.0)	0.02
Adjusted one year risk in %**						
Death	3.4	2.8	3.3	1.9	3.8	0.08
Death/MI	4.4	3.5	4.3	2.4	4.5	0.04
TVR	1.1	1.6	0.9	1.0	2.0	0.27
MACE	5.7	4.9	5.4	3.3	6.4	0.03

*MI* myocardial infarction, *TVR* target vessel revascularisation, *MACE* major adverse cardiac events (death/MI/TVR). * Number of events are counts, and percent in parentheses is the unadjusted one-year risk (survival probability calculated from the Kaplan-Meier estimate at 1 year. P-value is from log-rank test across years).

** Adjusted one-year risk (%) is the survival probability calculated from the fitted multivariate Cox model for 2000-2004 that includes calendar year, age, Charlson score, gender, index principal discharge diagnosis, type of index admission, type of hospital, number of vessels treated and type of CARP (only for All CARPs data). P-value is from Type 3 Wald test for calendar year from the fitted Cox model.

Table 3 shows adjusted HRs for one-year outcomes/endpoints from Cox regression models. As in Table 2, the “risk” or hazard of death and death/MI did not change by calendar year, but there was a marked decline in hazard for TVR between 2000-2002 and 2003-2004. The risk of death and death/MI increased with age, Charlson score, emergency admission and number of vessels treated. The risk of TVR increased with Charlson score, emergency admission, and number of vessels treated, was borderline for age and principal diagnosis of MI, and was reduced for admission to public hospitals. The risk of death within 12 months was lower following PCI than CABG ((HR 0.75, 95% confidence interval (CI) 0.60, 0.94). However, the risk of TVR or MACE was higher in PCI than CABG (HR 8.15, 95% CI 6.05, 10.97; and HR 2.37, 95% CI 2.03, 2.78, respectively).

**Table 3:** Fitted multivariate Cox model for one-year outcomes/endpoints in the total coronary artery revascularisation cohort from 2000 to 2004

**Variable**	**Adjusted hazard ratios (95% CI)**			
	**Death**	**Death/MI**	**TVR**	**MACE**
Year of procedure:*2000	1.0	1.0	1.0	1.0
2001	0.95 (0.72, 1.27)	0.90 (0.72, 1.13)	0.95 (0.78, 1.16)	0.91 (0.78, 1.07)
2002	1.03 (0.79, 1.36)	0.96 (0.77, 1.20)	0.72 (0.58, 0.89)	0.83 (0.70, 0.97)
2003	0.87 (0.66, 1.15)	0.80 (0.64, 1.00)	0.55 (0.44, 0.69)	0.64 (0.54, 0.75)
2004	1.19 (0.92, 1.55)	1.05 (0.85, 1.29)	0.54 (0.43, 0.67)	0.74 (0.63, 0.87)
Age at index admission	1.06 (1.05, 1.07)	1.04 (1.03, 1.05)	1.00 (1.00, 1.01)	1.02 (1.01, 1.02)
Gender (male) *	0.90 (0.75, 1.09)	0.92 (0.79, 1.08)	0.92 (0.78, 1.08)	0.91 (0.81, 1.03)
Charlson score at index admission	1.31 (1.27, 1.34)	1.28 (1.25, 1.31)	1.06 (1.02, 1.10)	1.19 (1.17, 1.22)
Index principal diagnosis (MI) *	1.24 (1.01, 1.52)	1.13 (0.96, 1.34)	0.83 (0.69, 1.00)	1.00 (0.88, 1.14)
Index admission type (emergency) *	2.04 (1.65, 2.52)	1.88 (1.58, 2.23)	1.18 (1.00, 1.40)	1.45 (1.28, 1.65)
Index hospital type (public) *	1.17 (0.96, 1.43)	1.25 (1.06, 1.47)	0.82 (0.71, 0.96)	0.99 (0.89, 1.11)
Number of vessels treated (multivessel disease) *	1.35 (1.09, 1.66)	1.24 (1.04, 1.47)	1.47 (1.24, 1.76)	1.35 (1.18, 1.54)
CARP type (PCI) *	0.75 (0.60, 0.94)	1.18 (0.97, 1.43)	8.15 (6.05, 10.97)	2.37 (2.03, 2.78)

*95% CI* 95% confidence interval, *MI* myocardial infarction, *TVR* target vessel revascularisation, *MACE* major adverse cardiac events (death/MI/TVR), *CARP* coronary artery revascularisation procedure, *PCI* percutaneous coronary intervention.

* Baseline levels are year 2000, female, no MI as principal discharge diagnosis in index admission, booked index admission, index admission in a private hospital, single vessel disease and bypass surgery (CABG).

Age (years) and Charlson score are continuous variables.

## Discussion

Age standardised rates of CARP have steadily increased in Western Australia since 1980. Bare metal stents were introduced into Western Australia in 1988 as a bailout strategy for complicated balloon angioplasty and their use increased through the early 1990s, becoming a primary strategy for PCI by the mid-1990s. This increase in BMS use was associated with a period of accelerated growth in total CARP driven by an increase in PCI while there was a modest decline in rates of CABG. The introduction of DES in 2002 was similarly associated with a period of accelerated growth in CARP driven by the near complete substitution of BMS with DES and further declines in rates of CABG. In each case, the introduction of new stent technology allowed some patients previously only suitable for CABG, and others only suitable for medical therapy, to be treated percutaneously with PCI.

At a population level, the introduction and widespread use of DES might improve outcomes by: (i) reducing adverse events after PCI; (ii) allowing treatment of those previously unsuitable for revascularisation; and (iii) allowing percutaneous treatment of high-risk surgical patients. However, it is also possible that outcomes could deteriorate due to: (i) increased stent thrombosis, MI and death; (ii) increased bleeding related to dual antiplatelet therapy; and (iii) increased adverse events in those treated percutaneously who may have been better treated surgically.

To explore these issues further, we assessed the one year outcomes for all CARP (PCI and CABG) over the period before and after the introduction of DES into Western Australia. We have shown that in the total CARP population: (i) there were no temporal changes in the risk of major clinical endpoints (death or death/MI), and (ii) there was a marked reduction in the one-year risk of TVR and MACE over time for the total CARP population, driven by a significant reduction in TVR in the PCI cohort with little change in the CABG cohort. These results support the effectiveness and safety of DES in reducing adverse events following PCI in a real world setting. In addition, the increase in total CARP rates and reduced proportion treated surgically was not associated with an increase in death or MI, further supporting the safety of this change in practice of coronary revascularisation.

Late stent thrombosis with DES remains a controversial issue. Previous meta-analyses have suggested increasing rates of stent thrombosis/MI and death in patients receiving DES [12,13]. However, much of this has been refuted by careful analysis of larger randomized groups as well as data from registries [14-16]. In our study, the stable risks of death and MI following PCI do not support a clinically significant increase in risk of stent thrombosis associated with the change from BMS to DES. The long-term follow-up of our study cohort, which is currently in progress, will allow us to determine if there is any evidence to suggest reduced long-term safety of DES, especially with outcomes of MI and stent thrombosis.

We also observed a lower adjusted risk of death in the PCI cohort compared to the CABG cohort in the Cox regression models. The higher 28-day and one-year mortality in the CABG cohort compared to PCI is not unexpected in this non-randomised study and likely reflects the combination of procedural risk and selection of patients with more diffuse disease and complex comorbidities for surgical revascularisation. The lower proportion of major clinical outcomes (but not TVR) in the CABG cohort in 2003 (the year following the introduction of DES) is not easily explained by our data. However, we cannot rule out variation in the proportion of cases per surgeon in that year, changes to the indications for surgery, or a shift from CABG to DES for high-risk cases.

The introduction and rapid uptake of DES has led to a significant decrease in TVR over the study period in the PCI group and all CARP population. The main driver of TVR is restenosis of the treated vessel. The threshold at which angiographic restenosis leads cardiologists to recommend a TVR is not expected to have changed over the study period, so it is likely that the observed reduction in TVR represents a reduction in restenosis. The significance and importance of TVR as an endpoint, especially compared with death and MI, has been debated widely. However, data from the SIRIUS trial showed that patients who have clinically driven TVR have worse long-term outcomes of death or MI following the TVR [17]. Therefore its economic, social and clinical impact should not be underestimated.

In this observational study, we cannot be certain that there is a causal relationship between the changing coronary revascularisation strategies and the observed reduction in TVR and stability in rates of death and MI. However, a unique aspect of our study cohort was the rapid transition from BMS to DES during 2002, which resulted in a decline in the use of BMS from 100% in 2000-2001 to less than 10% in 2003-2004, with a reciprocal increase in the use of DES. While this rapid uptake of DES was associated with changes in case selection for PCI which could have affected outcomes, bias due to temporal changes in other medical treatment and case severity would have been minimised although not totally excluded. We have attempted to adjust for such effects by including calendar year in our multivariate regression models, in addition to demographic and clinical factors known to affect outcomes. However, we acknowledge that since patients undergoing revascularisation in 2000-2004 were not randomly assigned to a treatment year there may be unknown or unmeasured confounders that might introduce bias in the calendar year comparisons.

Although there are limitations reflecting complete capture of past medical history in calculating a comorbidity score from administrative data, this limitation would not bias one group over another in our study because we investigated the total cohort of coronary artery procedures. All patients in this cohort presented with coronary heart disease and detection of risk factors and reporting of past medical history would be consistent for each person in the cohort. Furthermore, the comorbidities recorded in our administrative dataset are diagnoses made by doctors as recorded in medical notes. The Charlson score as a single measure of comorbidity had an area under the ROC curve of about 70% (range 65.6-75.5) for predicting one-year mortality [18], with the predictive ability improving after adjusting for age and sex [18], as we have done in our regression models.

A major strength of our study is the inclusiveness of our cohort (all index CARP) and complete data on outcomes and endpoints in the follow-up period from both clinical and administrative data sources. Pivotal to this is our linkage of procedural data to routinely collected hospital admission and mortality data which allows identification of co-morbid conditions and accurate follow-up of patients. There is no reason to suggest that our findings are only applicable to the Australian population. The revascularisation technologies in use in Australia over that time period, as well as in current times, are the same as were available in other countries. Therefore, the results of our population-based analysis are applicable in other jurisdictions throughout the world. The transition from BMS to DES was universal during this period and therefore this analysis is not only a reflection of an Australian population, but should also be a reflection of practice throughout the world.

## Conclusions

In summary, changing coronary revascularisation strategies related to the introduction of DES were not associated with changes in one-year risk of major clinical endpoints (death or death/MI), but were associated with a significant reduction in the risk of MACE, driven entirely by a reduction in TVR after PCI. This real world study supports the effectiveness of DES in reducing repeat procedures in the total CARP population without increasing the risk of death or MI. Our current very long-term follow-up of the study cohort will allow analysis well beyond the early risk period of one year, and provide population-based evidence of any long-term benefits of care and cost-effectiveness in the revascularisation population.

## Abbreviations

BMS: Bare metal stents; CABG: Coronary artery bypass graft; CARP: Coronary artery revascularisation procedures; DES: Drug eluting stents; HMDC: Hospital Morbidity Data Collection; ICD: International Classification of Diseases; MACE: Major adverse cardiac events; MI: Myocardial infarction; PCI: Percutaneous coronary intervention; PTCA: Percutaneous transluminal coronary (balloon) angioplasty; TVR: Target vessel revascularisation.

## Competing interests

The authors declare that they have no competing interests.

## Authors’ contributions

FS was the day-to-day coordinator of the study, assisted in the study design, developed the data collection database, maintained the Endnote library of references, carried out all of the analyses, was the lead author for the methods and results sections of the manuscript, and provided major contributions to the background and discussion. JR was the lead cardiologist who had the original idea for the study and obtained funding, with major contributions to the study design, data collection and analytical methods, interpreted the results and made significant contributions to the manuscript drafts. MH was the lead cardiovascular epidemiologist in the study with major contribution to the study design and data collection methods, and significant contribution in preparing the discussion section of the manuscript. MN is a cardiologist who assisted in interpreting the data and made major contributions in preparing the discussion section of the manuscript. MK is a biostatistician who contributed to the study design and analytical methods, supervised the analyses and assisted in interpreting the results. PB supervised and participated in the data collection for PCI procedures from private hospitals. EW, RH, BH and MM are cardiologists (representing mostly private hospitals) who assisted in the logistics of data collection and contributed to interpretation of the results. MN, RL and IG are cardiothoracic surgeons (representing all of the hospitals where CABG is performed) who provided interpretation of the results from the surgical perspective, and assisted in the logistics of data collection for CABG procedures. TB is a cardiovascular epidemiologist who assisted in interpreting the results. All authors reviewed and provided comments for the draft manuscripts, and read and gave approval for release of the final manuscript.

## Pre-publication history

The pre-publication history for this paper can be accessed here:

http://www.biomedcentral.com/1471-2261/13/47/prepub

## Supplementary Material

Additional file 1 **Supplementary analysis.** Analysis of one-year outcomes/endpoints in patients with: (i) left main coronary artery disease in the PCI cohort 2000-2004; (ii) multi-vessel disease 2000-2004. Click here for file
