# Supplementary material for: Impact of the introduction of drug eluting stents on clinical outcomes in patients undergoing percutaneous and surgical coronary artery revascularisation procedures in Western Australia
Source: BMC Cardiovasc Disord. 2013 Jul 5;13:47. doi: 10.1186/1471-2261-13-47 (PMC3704943; doi:10.1186/1471-2261-13-47)
Supplement: Additional file 1 — Supplementary analysis. Analysis of one-year outcomes/endpoints in patients with: (i) left main coronary artery disease in the PCI cohort 2000-2004; (ii) multi-vessel disease 2000-2004. [file 1471-2261-13-47-S1.pdf]

Impact of the introduction of drug eluting stents on clinical outcomes in patients undergoing percutaneous and surgical coronary artery revascularisation procedures in Western Australia

Supplementary Analysis

The PCI and CABG cohorts included patients with left main coronary artery disease and those with multi-vessel disease. Left main disease was present in only 120 PCI cases, with 17 deaths and 33 MACE (death/MI/TVR) within 12 months of the index procedure. The number of outcome events per year from 2000-2004 was therefore too small to perform a reliable Cox regression analysis of left main disease in the PCI cohort (see Table S1). The clinical data we had for the CABG cohort were less complete in recording left main disease (left main coronary arteries are usually bypassed rather than grafted), and we could not separate LM disease from multi-vessel disease in the CABG cohort, so we were not able to do a reliable subgroup analysis of left main disease in the CABG cohort.

However, we do have complete data for multi-vessel disease in both the PCI and CABG cohorts. Trends in adverse outcomes/endpoints (death, MI, TVR) within 12 months of index CARP in patients with multi-vessel disease are shown in Table S2 for each type of procedure, including estimates of one-year risk from Kaplan-Meier curves (unadjusted) and Cox regression models (adjusted). Trends in 12-month outcomes were similar to those of the total PCI and CABG cohorts reported in Table 2 of the manuscript, with higher unadjusted and adjusted risks seen in the PCI subcohort with multi-vessel disease, and slightly higher risks in the CABG subcohort with multi-vessel disease.

Table S1 Counts and proportions of one-year outcomes/endpoints in patients with left main coronary artery disease in the PCI cohort 2000-2004

**LM disease PCI cohort**	**2000**	**2001**	**2002**	**2003**	**2004**	**Total**
LM cases	18 (1.1)	13 (0.8)	18 (1.0)	32 (1.5)	39 (1.7)	120 (1.2)
12-mth death	0	2 (15.4)	4 (22.2)	5 (15.6)	6 (15.4)	17 (14.2)
12-mth death/MI	0	2 (15.4)	5 (27.8)	6 (18.8)	7 (18.0)	20 (16.7)
12-mth TVR	4 (22.2)	2 (15.4)	3 (16.7)	1 (3.1)	4 (10.3)	14 (11.7)
12-mth MACE	4 (22.2)	4 (30.8)	7 (38.9)	7 (21.9)	11 (28.1)	33 (27.5)

LM, left main coronary artery; PCI, percutaneous coronary intervention; MI, myocardial infarction; TVR, target vessel revascularisation; MACE, major adverse cardiac events (death/MI/TVR).

Table S2 One-year outcomes/endpoints by type of revascularisation in patients with multi-vessel disease 2000-2004

	2000	2001	2002	2003	2004	p-value
**ALL CARPS (PCI, CABG)**						
**Number of events (unadjusted one-year risk in %)***						
Death	54 (6.1)	49 (5.6)	50 (5.7)	31 (3.4)	53 (5.5)	0.07
Death/MI	68 (7.6)	58 (6.7)	60 (6.8)	47 (5.1)	66 (6.9)	0.26
TVR	38 (4.4)	37 (4.4)	35 (4.2)	37 (4.1)	44 (4.8)	0.95
MACE	100 (11.2)	87 (10.0)	90 (10.3)	75 (8.1)	105 (11.0)	0.18
**Adjusted one year risk in %****						
Death	3.8	3.7	3.5	2.3	3.7	0.19
Death/MI	5.4	4.9	4.7	3.7	4.7	0.36
TVR	3.0	2.9	2.6	2.0	2.1	0.21
MACE	9.6	8.7	8.5	6.1	7.6	0.03
**PCI**						
**Number of events (unadjusted one-year risk in %)***						
Death	9 (4.6)	11 (5.3)	12 (5.3)	13 (3.9)	26 (6.2)	0.68
Death/MI	13 (6.8)	18 (8.6)	18 (7.9)	26 (7.8)	36 (8.6)	0.93
TVR	29 (15.4)	29 (14.3)	30 (13.5)	31 (9.5)	33 (8.3)	0.03
MACE	38 (19.5)	41 (19.6)	43 (18.9)	48 (14.4)	64 (15.3)	0.28
**Adjusted one year risk in %****						
Death	3.4	3.7	2.9	2.4	3.4	0.82
Death/MI	5.3	6.9	5.5	5.6	6.1	0.93
TVR	14.2	13.9	13.4	9.1	8.3	0.06
MACE	18.4	19.0	17.9	13.1	14.2	0.18
**CABG**						
**Number of events (unadjusted one-year risk in %)***						
Death	45 (6.5)	38 (5.8)	38 (5.9)	18 (3.1)	27 (5.0)	0.07
Death/MI	55 (7.9)	40 (6.1)	42 (6.5)	21 (3.6)	30 (5.6)	0.03
TVR	9 (1.4)	8 (1.3)	5 (0.8)	6 (1.0)	11 (2.1)	0.37
MACE	62 (8.9)	46 (7.0)	47 (7.2)	27 (4.6)	41 (7.6)	0.05
**Adjusted one year risk in %****						
Death	3.8	3.5	3.7	2.1	3.4	0.29
Death/MI	5.0	4.0	4.4	2.6	4.0	0.16
TVR	1.3	1.2	0.8	1.0	2.0	0.41
MACE	6.3	5.2	5.4	3.7	6.0	0.19

MI, myocardial infarction; TVR, target vessel revascularisation; MACE, major adverse cardiac events (death/MI/TVR); PCI, percutaneous coronary intervention; CABG, coronary artery bypass graft surgery.

* Number of events are counts, and percent in parentheses is the unadjusted one-year risk (survival probability calculated from the Kaplan-Meier estimate at 1 year. P-value is from log-rank test across years).

** Adjusted one-year risk (%) is the survival probability calculated from the fitted multivariate Cox model for 2000-2004 that includes calendar year, age, Charlson score, gender, index principal discharge diagnosis, type of index admission, type of hospital and type of CARP (only for All CARPs data). P-value is from Type 3 Wald test for calendar year from the fitted Cox model.
